# Post-graduate medical education in public health: the case of Italy and a call for action

**DOI:** 10.1186/s40985-017-0069-0

**Published:** 2017-10-24

**Authors:** Anna Odone, Gaetano Pierpaolo Privitera, Carlo Signorelli, Board of Directors of the Italian Postgraduate Schools of Hygiene and Public Health, Marcello Mario D’Errico, Michele Quarto, Maria Pia Fantini, Francesco Donato, Paolo Contu, Marina Marranzano, Aida Bianco, Tommaso Staniscia, Giovanni Gabutti, Paolo Bonanni, Roberto Gasparini, Leila Fabiani, Isa Picerno, Giancarlo Maria Cesana, Mirella Maria Pontello, Carlo Signorelli, Paola Borella, Ida Torre, Francesco Attena, Vincenzo Baldo, Alessandra Casuccio, Gabriele Pelissero, Fabrizio Stracci, Gaetano Pierpaolo Privitera, Gianfranco Damiani, Paolo Villari, Elisabetta Franco, Mario Capunzo, Ida Mura, Gabriele Messina, Roberta Siliquini, Maria Parpinel, Gabriele Romano

**Affiliations:** 1 Department of Medicine and Surgery, University of Parma, Parma, Italy; 2 Department of Translational Research and New Technologies in Medicine and Surgery, University of Pisa, Pisa, Italy; 3 School of Medicine, University Vita-Salute San Raffaele, Milan, Italy

**Keywords:** Public health, Hygiene and preventive medicine, Training, Education, Survey

## Abstract

Public health technical expertise is of crucial importance to inform decision makers’ action in the field of health and its broader determinants. Improving education and training of public health professionals for both practice and research is the starting point to strengthen the role of public health so that current health challenges can be efficiently tackled. At the Association of Schools of Public Health in the European Region (ASPHER) Deans’ & Directors’ 2017 Annual Retreat, we presented the structure and management of public health training system in Italy, and we reported recent data on Italian public health specialists’ educational experience, employment opportunities and job satisfaction. Public health training in Italy is implemented in the context of the post-graduate medical education residency programme in Hygiene and Preventive Medicine, delivered by 34 University-based Schools of Public Health. We report relatively high employment rates across the county and wide spectrum of career opportunities for young public health specialists. However, job security is low and training expectations only partially met. We call upon other Schools of Public Health to scale up the survey within the broad ASPHER community in a shared and coordinated action of systematically collecting useful data that can inform the development of public health education and training models, their implementation and fruitful interaction with population health, health systems and services.

## The scope for investing in public health training

In times where health systems’ sustainability is undermined by populations’ ageing, ongoing economic crises, increased burden of chronic diseases and healthcare costs, national and international health authorities have underlined the importance of investing in public health policies [1–4]. As stated by the World Health Organization, to tackle current health challenges, the role of public health has to be strengthened throughout all its domains, including public health governance, workforce development, advocacy and research [2]. We firmly believe effective public health education and training is the fundamental starting point for this to happen, in a balanced triangle where public health *education* meets public health *practice* needs, supports public health workforce development and is informed by *research*. As a matter of fact, public health technical expertise is of crucial importance to inform decision makers’ action in the field of health and its broader determinants.

Public health training’s opportunities and programmes in different countries differ by structure, contents, curricular pathway, entry requirements and quality and are usually shaped and managed on the basis of national education systems and national health services’ features. At the Association of Schools of Public Health in the European Region (ASPHER) Deans’ & Directors’ Annual Retreat hold on May 31–June 2, 2017, we presented the case of Italy, describing the Italian public health training model and reporting the findings of a recent survey we conducted to explore Italian public health specialists’ training experience, employment opportunities and job satisfaction.

## The Italian model of public health training

Public health workforce employed in the Italian National Health service (INHS) mainly consists of public health specialist medical doctors; a much more limited share includes public health nurses and veterinaries, occupational physicians, prevention technicians and health care assistants. This medically centred distribution reflects the structure of public health training in Italy, which is embedded in the post-graduate medical education system. The post-graduate medical education system consists of 50 different residency programmes in clinical, surgical and non-clinical sectors. Non-clinical residency programme includes: (i) Hygiene and Preventive Medicine, (ii) Occupational health, (iii) Legal medicine and (iv) Medical statistics.

**Fig. 1:**
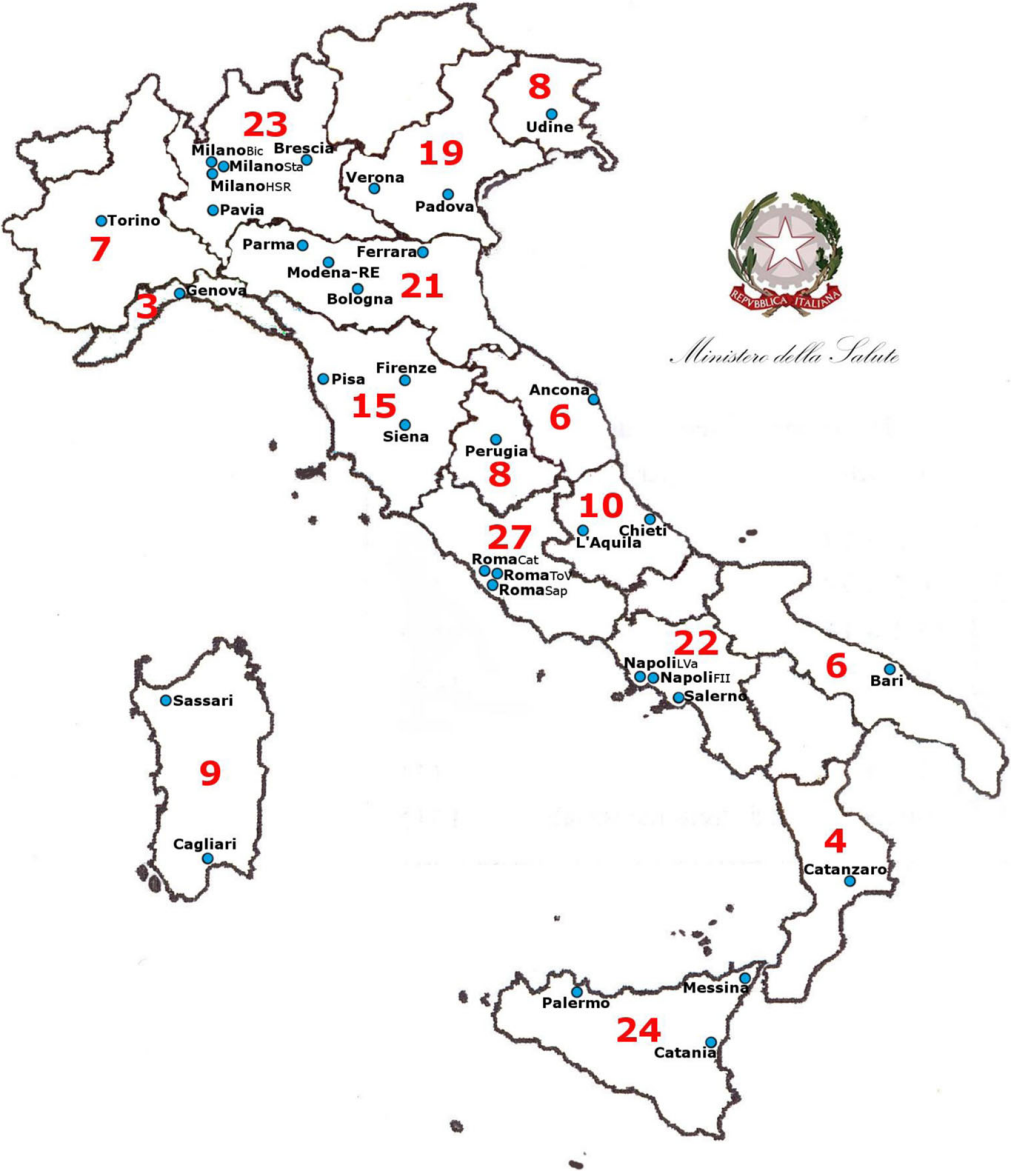
Hygiene and Preventive Medicine residency programme available contracts, by School of Public Health and by region, 2016

The post-graduate medical residency in “Hygiene and Preventive Medicine” (School of Public Health—SPH from now on) is currently a 4-year training programme, delivered by University-based schools accredited jointly by the Italian Ministry of Education and the Ministry of Health. There are 34 SPHs in Italy (Fig. 1), located in all 20 but 3 Italian regions. All SPHs’ Directors are University Professors of Hygiene and Public Health and jointly constitute the Board of Directors of the Italian Schools of Public Health, a national Board hosted by the Italian Society of Hygiene, Preventive Medicine and Public Health (SItI) and ASPHER member since 2015.

Every year, a Ministerial Decree defines the number of available SPH contracts (around 200 per year), as well as the national entry requirements and application process rules. The application process is managed at the national level, acceptance being based on academic performance. In 2009, acknowledging the need of skilled public health professionals, the PH residency programme was recognized as ‘strategic for the Italian National Health Service’ and awarded additional contracts. Available contracts are funded by the Ministry of Health, and distributed across Italian Regions on the basis of the estimated INHS staff needs (In 2016 there were 212 available contracts, distributed in different SPHs and different Regions, as illustrated in Fig. 1).

With the aim of aligning the post-graduate medical education system in Italy to the best European standards, subsequent Ministerial decrees [5–7] have defined and progressively updated the PH residency programme curricula and standards. In particular, in 2013, a national-level management of the residency programmes’ application process was introduced, as opposed to single universities local-level management, this to promote meritocracy and transparency in the selection process in the whole country [8]; in 2015 a decree jointly issued by the Ministry of Education and the Ministry of Health reduced the SPH programme from 5 to 4 years [9]. The most recent decree, published in June 2017 [10], lists SPHs accreditation criteria in terms of structural and organizational standards, professional development requirements, included core competencies, staff and faculty capacity needs.

Scant data are available on standards’ implementation in different settings and on residents’ training experience in the field. Previous surveys conducted among Italian PH residents [11–15] reported high heterogeneity of training experiences both within and between SPHs with regard to courses’ offer, implemented professional activities, extra-curricular opportunities, training experience rating and—most importantly—training programmes' adherence to Ministerial standards.

## Survey results on employment opportunities, job satisfaction and educational experience in public health

Building up on previous surveys' findings and with the aim of collecting recent data useful to plan and implement improvements of public health training in Italy, we conducted a nationally representative cross-sectional study among PH residents who specialized in the last 2 years (between October 2014 and July 2016). Specific objectives were to assess their training experience, employment opportunities and current job satisfaction. The online survey was carried out with the support of SItI, and the survey tool was built and piloted by a scientific committee of the Board of Directors of the Italian Schools of Public Health. The target population was contacted via email through the national PH *alumni* mailing list and administered the questionnaire which was anticipated by a letter from SItI President asking for collaboration and presenting the general aims and specific objectives of the survey. We were able to contact 94.4% (255/270) of the target population, 91% accessed the questionnaire. The overall response rate was 49%, with homogeneous geographical distribution.

**Fig. 2:**
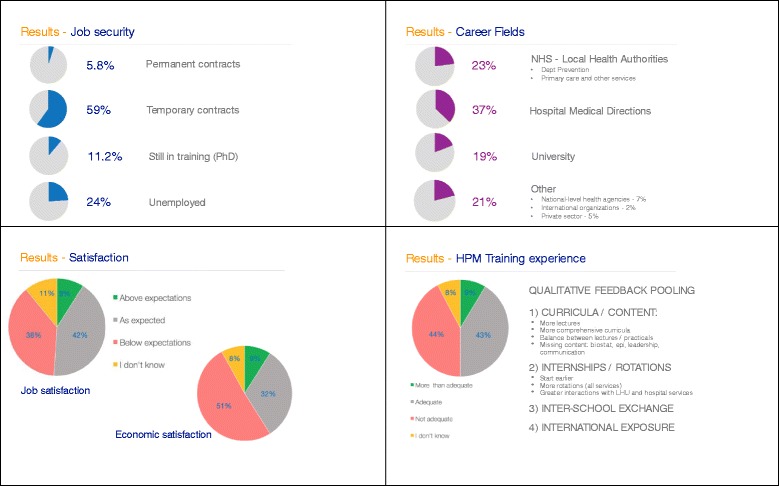
Survey results: career opportunities, job security, satisfaction and training experience in young Italian public health specialists, 2016

From the picture we took, recent PH specialists in Italy have an average of 35.2 years (DS 4.8 - range 29–60) and are females in 67.2% of cases. More than two thirds (76%) of them are employed, with a neat North-South employment rate gradient (77% in the North, 68% in central Italy, and 60% in the South). When stratifying data by graduation cohort, 2 years after graduation 93.5% of public health specialist is employed. However, only 5.8% of young PH specialists have a permanent contract, 59% holding different types of temporary contracts and 11.2% opted to stay in training pursuing a PhD degree (Fig. 2). National mobility is relatively low with 80% of employed subjects working in the same region where they attended the PH residency programme, the percentage being the lowest in the South of Italy where 32% of respondents declared to have moved, mostly to the North to work. Low national mobility might be linked to the fact that respondents were enrolled in the PH residency programme when the application process was still managed at the local level and might increase in the near future, since it is now managed at the central national level.

Public health specialists in Italy mostly fill positions within the INHS. As emerges from our survey, the large majority (37%) works in Hospitals’ Medical Directions, of which 20% in the public sector and 17% in INHS-accredited private hospitals or clinics(Fig. 2). Twenty-three percent of subjects work for the INHS at the Local Health Authority (LHA) level, either in Departments of Prevention, in the primary care sector, and other local public health and preventive services; 7% are employed in regional or national-level health agencies. Less than 20% of PH specialists remain in the academia occupying research positions, 5% are employed in the private sector (i.e. pharmaceutical companies and consulting agencies) and 2% join international organizations or research centres abroad. Job and economic satisfaction are both reported to be ‘above expectations’ in only 9% of cases, while ‘below expectations’ in, respectively, 38% (job satisfaction) and 51% (economic satisfaction) of cases, percentage distributions varying by geographic area, satisfacyon being highest in the North of Italy.

Four percent of respondents hold another medical specialization degree in a clinical field, 10.4% complemented the residency training with a Master in Public Health (MPH), or a PhD (1.6%). Sixty-three percent of respondents reported to be SItI members and 28% to be members of the European Public Health Association (EUPHA). Of note, 44% of employed PH specialists judged his/her PH training experience partially inadequate in relation to current professional tasks. In particular, when asked what changes in PH training curricula would have helped them to be more competent and prepared in their jobs, the following suggestions consistently emerged (open questions’ feedback): a more comprehensive curriculum, incorporating strategic public health competencies (i.e. leadership and public speaking skills), more intensive training in quantitative data analysis (i.e. biostatistics and epidemiology), more credits awarded for frontal lectures and practical sessions. With regard to training internships and rotations, respondents declared they would have benefitted from an earlier start of professionalization activities in a wider variety of public services both in hospitals and local health authorities. Similarly, they felt they would have benefitted from a greater interaction with public health professionals during training, greater international exposure and deeper inter- and intra-SPHs exchange with both faculty and peers (detailed figures and data are available elsewhere [16]) (Fig. 2).

## Lessons learned and the way forward

As described, post-graduate public health training in Italy is essentially medically centred. This is in line with national public health workforce and job opportunities that still focus on medical profiles but move away from the international and European multidisciplinary approach to public health and task-shifting trends observed in recent years. Of interest, recent figures report that more than one third of health managers and leaders (hospitals’ and LHAs’ Director Generals) hold a public health specialty, one other third holding clinical specialties and the remaining one third holding other degrees, including law, economics and political sciences degrees [17].

Overall, we report relatively high employment rates for PH specialists in Italy and a wide variety of employment opportunities. However, our data show that young PH specialists are rarely contracted with permanent positions or tangible prospects of long-term career progressions; perceived job and salary satisfaction are only partially fulfilled. Public health training experience in the context of the PH residency programme varies within and between the 34 Italian Schools of Public Health. Both quantitative and qualitative findings from our survey have been shared and disseminated among the Board of Directors of the Italian Schools of Public Health to initiate a critical fruitful debate on how to improve public health programmes to better meet public health practice needs and take prompt and evidence-supported action.

## Conclusion

We (i) described the structure and management of public health training system in Italy, and we (ii) reported recent data on Italian public health specialists’ educational experience, employment opportunities and job satisfaction. Although the latter has been explored in other European and international settings, including Canada, France, Japan, the UK, and the USA [18, 19], no updated pooled figures are available in the literature. Since the EU passed in 2005, the Directive 2005/36/EC on the recognition of professional qualifications, automatic recognition of post-graduate medical specialties in PH applies to most Member States. This makes it of fundamental importance for public health educational standards to be met in different settings and according to different health systems and workforce development needs. As reported in other countries, gaps exist between current public health needs and the extent to which public health workers are trained [20–22]. In fulfilling its mission, ASPHER promotes activities which foster exchange of information and best practices among its members in an effort to achieve high standards of public health education and training across Europe [23]. On this basis, we call upon other Schools of Public Health to scale up the survey within the broad ASPHER community in a shared and coordinated action of systematically collecting useful data that can inform the development of public health education and training models, their implementation and fruitful interaction with population health, health systems and services.

## Abbreviations

HPM: Hygiene and Preventive Medicine
INHS: Italian National Health Service (Servizio Sanitario Nazionale)
LHA: Local Health Authorities (Aziende Sanitarie Locali)
PH resident: Resident physician in Public Health (specilizzando in igiene e medicina preventiva)
PH: Public Health
SItI: Italian Society of Hygiene, Preventive Medicine and Public Health
SPH: School of Public Health

## References

[1] World Health Organization. Regional Office for Europe. Action Plan for implementation of the European Strategy for the Prevention and Control of Noncommunicable Diseases 2012–2016. Copenhagen: WHO; 2012. ISBN 9789289002684. http://www.euro.who.int/__data/assets/pdf_file/0019/170155/e96638.pdf?ua=1.

[2] World Health Organization. Regional Office for Europe. The Case for Investing in Public Health. Copenhagen: WHO; 2014. http://www.euro.who.int/__data/assets/pdf_file/0009/278073/Case-Investing-Public-Health.pdf?ua=1.

[3] Signorelli C, Odone A, Bianco D, Di Vivo N, Bevere F (2016). Health expenditure for prevention in Italy (2006–2013): descriptive analysis, regional trends and international comparisons. Epidemiol Prev.

[4] Signorelli C, Ricco M, Odone A (2016). The Italian National Health Service expenditure on workplace prevention and safety (2006–2013): a national-level analysis. Annali di igiene : medicina preventiva e di comunita.

[5] Decreto Legislativo 30 dicembre 1992, n. 502. Riordino della disciplina in materia sanitaria, a norma dell’articolo 1 della L. 23 ottobre 1992, n. 421. GU 30 dicembre 1992, n. 305 (Suppl Ord.). Available at: http://www.gazzettaufficiale.it/eli/id/1994/01/07/094A0049/sg. Accessed 2 Oct 2017.

[6] Ministero dell’Istruzione, dell’Università e della Ricerca. Decreto ministeriale 1 agosto 2005. Riassetto delle Scuole di Specializzazione in Area Sanitaria. GU 5 novembre 2005, n. 285 – (Suppl Ord n. 176). Available at : http://attiministeriali.miur.it/anno-2005/agosto/dm-01082005.aspx. Accessed 2 Oct 2017.

[7] Decreto Legislativo 9 aprile 2008, n. 81. Testo coordinato con il Decreto Legislativo 3 Agosto 2009, n. 106- Testo unico sulla salute e sicurezza sul lavoro. Available at: http://www.lavoro.gov.it/documenti-e-norme/studi-e Statistiche/Documents/Testo%20Unico%20sulla%20Salute%20e%20Sicurezza%20sul%20Lavoro/Testo-Unico-81-08-Edizione-Giugno%202016.pdf. Accessed 2 Oct 2017.

[8] Decreto-legge 12 settembre 2013, n. 104 "Misure urgenti in materia di istruzione, universita' e ricerca". Available at http://www.gazzettaufficiale.it/eli/id/2013/11/11/13A09118/sg. Accessed 2 Oct 2017.

[9] Decreto Interministeriale 4 febbraio 2015 n. 68 "Riordino scuole di specializzazione di area sanitaria" Available at http://attiministeriali.miur.it/anno-2015/febbraio/di-04022015.aspx. Accessed 2 Oct 2017.

[10] Ministero dell'Istruzione dell'Università e della Ricerca, 2017. Decreto interministeriale recante gli standard, i requisiti e gli indicatori di attività formativa e assistenziale delle Scuole di specializzazione di area sanitaria n. 402. Avaialble at: http://www.miur.gov.it/-/decreto-interministeriale-recante-gli-standard-i-requisiti-e-gli-indicatori-di-attivita-formativa-e-assistenziale-delle-scuole-di-specializzazione-di-. Accessed 2 Oct 2017.

[11] Taietti D, Tirani M, Shahi E, Garavelli E, Nobile M, Cereda D (2015). Survey on professional training in three Italian. Post-Graduate Schools of Public Health. Hygiene Annals.

[12] Garavelli E, Marcantoni C, Costantino C, Tedesco D, Burrai V, Giraldi G (2014). Education and training among Italian postgraduate medical schools in public health: a comparative analysis. Hygiene Annals.

[13] Fara GM, Nardi G, Signorelli C, Fanti M (2005). Employment opportunities and education needs of physicians with specialty training in Hygiene and Preventive Medicine. Igiene e sanita pubblica..

[14] Fantini MP, Randazzo C, Rustico E, Tedesco D (2014). Residency in hygiene and preventive medicine: present and future. Epidemiol Prev.

[15] Costantino C, Cinquetti S, Garavelli E, Marcantoni C, Murru C, Pieroni G (2014). The key role of public health medical resident education for future public health challenges. Epidemiol Prev.

[16] Soncini F, Odone A, Lalic T, Miduri A, Paroni S, Vezzosi L, Privitera G, Signorelli. Sbocchi professionali, aspettative e gratificazioni dei neo-specialisti in Igiene e Medicina Preventiva: risultati di un'indagine nazionale. Ig San Pub 2017; 5 (in press). 29573384

[17] Cergas - Bocconi. Rapporto OASI 2013 Osservatorio sulle Aziende e sul Sistema sanitario Italiano. Available at: http://www.sossanita.it/doc/2014_01_OASI_Cap6_2013.pdf [accessed 29 June 2017].

[18] Peik SM, Mohan KM, Baba T, Donadel M, Labruto A, Loh LC (2016). Comparison of public health and preventive medicine physician specialty training in six countries: identifying challenges and opportunities. Medical teacher.

[19] Stein DH, Salive ME (1996). Adequacy of training in preventive medicine and public health: a national survey of residency graduates. Academic Med.

[20] Paccaud F, Weihofen A, Frank M (2013). Public Health Workforce in Switzerland: are public health workers lacking?. Int J Public Health.

[21] Berkenbosch L, Schoenmaker SG, Ahern S, Sojnaes C, Snell L, Scherpbier AJ (2013). Medical residents’ perceptions of their competencies and training needs in health care management: an international comparison. BMC medical education.

[22] D'Andrea E, Lucaroni F, Parente P, Damiani G, La Torre G, Mancinelli S (2016). What are the competencies that public health physician should have today? A proposal for a shared training program at three Hygiene and Preventive Medicine residency training schools in Rome (Italy). Igiene e sanita pubblica.

[23] The Association of Schools of Public Health in the European Region (ASPHER). Available at: http://www.aspher.org/. Accessed 2 Oct 2017.

